# Unintended Consequences: Nutritional Impact and Potential Pitfalls of Switching from Animal- to Plant-Based Foods

**DOI:** 10.3390/nu13082527

**Published:** 2021-07-23

**Authors:** Rachel Tso, Ciarán G. Forde

**Affiliations:** 1 Clinical Nutrition Research Centre (CNRC), Singapore Institute for Food and Biotechnology Innovation (SIFBI), Agency for Science, Technology and Research (A*STAR), Singapore 117599, Singapore; rachel_tso@sifbi.a-star.edu.sg; 2 Department of Physiology, Yong Loo Lin School of Medicine, National University of Singapore, Singapore 117593, Singapore; 3 Sensory Science and Eating Behaviour, Division of Human Nutrition and Health, Wageningen University and Research, P.O. Box 17, 6700 AA Wageningen, The Netherlands

**Keywords:** flexitarian, vegetarian, vegan, plant-based meat alternatives, nutrient intakes

## Abstract

Consumers are shifting towards plant-based diets, driven by both environmental and health reasons. This has led to the development of new plant-based meat alternatives (PBMAs) that are marketed as being sustainable and good for health. However, it remains unclear whether these novel PBMAs to replace animal foods carry the same established nutritional benefits as traditional plant-based diets based on pulses, legumes and vegetables. We modelled a reference omnivore diet using NHANES 2017–2018 data and compared it to diets that substituted animal products in the reference diet with either traditional or novel plant-based foods to create flexitarian, vegetarian and vegan diets matched for calories and macronutrients. With the exception of the traditional vegan diet, all diets with traditional plant-based substitutes met daily requirements for calcium, potassium, magnesium, phosphorus, zinc, iron and Vitamin B12 and were lower in saturated fat, sodium and sugar than the reference diet. Diets based on novel plant-based substitutes were below daily requirements for calcium, potassium, magnesium, zinc and Vitamin B12 and exceeded the reference diet for saturated fat, sodium and sugar. Much of the recent focus has been on protein quality and quantity, but our case study highlights the risk of unintentionally increasing undesirable nutrients while reducing the overall nutrient density of the diet when less healthy plant-based substitutes are selected. Opportunities exist for PBMA producers to enhance the nutrient profile and diversify the format of future plant-based foods that are marketed as healthy, sustainable alternatives to animal-based products.

## 1. Introduction

Global concerns around the consumption of animal products and their adverse effects on health and the environment have led to significant growth in the plant-based protein space, particularly for new products to replace traditional meat and dairy [1,2,3,4]. This increased demand for non-animal protein has resulted in more consumers declaring to be “flexitarian” or choosing to reduce meat, dairy and eggs in favour of more plant-based foods to benefit the environment, improve health or both [5]. Consumer market insights suggest that from 2019 to 2020, as many as 5 million consumers in the United States shifted to avoid meat completely, becoming either vegetarians or vegans [6,7]. Non-animal-based proteins include vegetable sources, plant-based meat alternatives (PBMAs) and less commonly consumed sources such as algae, insects and cultured meat [8]. The PBMA market is sizable and growing, valued at USD 4.3 billion in 2020 and projected to reach USD 8.3 billion by 2025 [9]. In addition to improved sustainability, PBMAs are marketed for their nutritional benefits [10,11], driving an increase in their consumption for health reasons [2,3]. However, the health benefits of novel PBMAs—a product category with diverse formulations and nutritional compositions—to replace animal foods long-term remain unclear, due to a lack of longitudinal evidence and randomised controlled trials. There has been the suggestion of a “health halo” around these products, where established health benefits of vegetarian and vegan diets are being conflated with positive messaging around animal welfare, sustainability and the environment for many of the newer PBMA products [8,12]. An additional concern is the current focus on promoting plant-based foods of “unhealthy” product categories and formats such as plant-based burgers, nuggets, meatballs and sausages, which may increase consumption of so-called “junk foods” [8,13].

A concern for many consumers seeking to decrease their meat intake and switch to plant-based diets is potentially compromising their protein intake in terms of quality and quantity [14,15]. To add to consumers’ uncertainty, less is known about the wider impact of substituting animal foods with plant-based products—particularly novel ones—on intakes of micronutrients and public health-sensitive nutrients (fat, sugar and sodium). Much more is known about the health benefits of omitting meat in favour of traditional plant sources such as legumes, with extensive evidence demonstrating lowered risks of cardiovascular disease, diabetes, cancer and obesity for vegetarians and vegans [16,17,18,19]. Even partially reducing meat while increasing healthful plant-based foods has been shown to be beneficial and is associated with lower risks of disease and mortality [4,20,21]. The 2020–2025 Dietary Guidelines for Americans recommend vegetarian diets as one of three healthful dietary patterns [22], the other two being the Healthy U.S.-Style Dietary Pattern and the Healthy Mediterranean-Style Dietary Pattern [22]. The Mediterranean diet is a well-studied example of a feasible plant-based eating pattern low in red meat and high in unsaturated fat intake [4]. It is associated with numerous health benefits including reduced incidence of cardiovascular disease and greater longevity [23,24] while having a lower environmental impact compared to a typical omnivorous Western diet [25].

Frequent red and processed meat intake is linked to greater risks of cardiovascular disease and cancer [26,27]; however, the World Cancer Research Fund highlights that this does not mean all consumers must completely avoid meat. This is because meat presents a valuable source of protein, iron, zinc and Vitamin B12 [28]. Guidelines recommend a maximum of three portions of red meat weekly, while also limiting or avoiding processed meat [28]. There is also evidence that the relationship between meat intake and risk of stroke differs by type of meat. A systematic review and meta-analysis of total, red, processed and white meat consumption found total stroke incidence to be lowest for consumers of white meat [29]. Whether novel PBMAs and traditional plant-based foods make nutritionally equivalent animal food substitutes remains to be seen.

### 1.1. Protein and Nutrient Concerns between Animal- and Plant-Based Diets

Animal and plant proteins are both nutritious, and protein quality and quantity are often not compromised when switching to well-designed plant-based diets [30]. This applies whether meat and dairy are reduced or completely excluded in favour of nutritious plant-based alternatives. Animal products provide an important source of nutrients and have been described as “complete” protein sources with “high biological value” since they contain all nine essential amino acids [31]. Many plant foods do not contain all essential amino acids and are termed “incomplete” protein sources [31]. The terms “complete”, “incomplete”, “high biological value” and “low biological value” have been challenged and termed “misleading” in relation to plant protein because they reflect the quantity and quality of essential amino acids consumed in a single serving, but do not account for plant protein blends or the overall protein quality of the diet to meet requirements [30,32]. Most plant-based foods are consumed in combination and from a variety of sources such that plant-based diets adequately meet requirements for all essential amino acids in a calorie-sufficient diet [33]. Furthermore, evidence is emerging on the equivalence of animal and plant protein sources in sustaining lean muscle mass and strength in extended feeding trials [34]. In this regard, it is possible to achieve equivalent protein quality and quantity through the partial or complete removal of animal products in favour of a plant-based diet. What is less well understood is the impact of such a large dietary shift in consumption patterns on intakes of other dietary components, particularly micronutrients.

On-pack nutritional composition represents the nutrients of the unprepared product and often does not reflect actual nutrients consumed when products require further preparations that influence their final composition. This is especially true for most novel plant-based products. Whereas many traditional vegetarian dishes require minimal oil and salt such as lentil stews and stir-fried tofu, by contrast, most of the current PBMA products are sold in formats such as burgers, sausages or nuggets that require preparation with more oil (i.e., frying) and salt and are often consumed with nutrient-poor sides, condiments and beverages. Regularly consuming these products could potentially lead to higher calorie, fat and salt intakes. Similarly, non-dairy milks targeted towards vegetarians or vegans are often high in added sugar [35], while many vegetarian and vegan spreads, snacks and desserts have high levels of salt and fat. If PBMAs or vegetarian- or vegan-friendly products have poor nutritional profiles but are sold under the pretence of better health or nutritional equivalence to natural plants, consumers who adopt them to support a flexitarian, vegetarian or vegan diet may unintentionally consume nutrient-poor diets that are higher in public health-sensitive nutrients.

### 1.2. Case-Study: A Comparison of Omnivore, Flexitarian, Vegetarian and Vegan Diets

Similar to all diet and lifestyle changes, switching to plant-based substitutes for meat can be beneficial or not for health. In the absence of clear data on the nutritional impact of such a change, the current case study sought to compare the health impact of substituting animal products (i.e., meat, dairy and eggs) for both traditional and emerging non-animal alternatives. We chose a standard omnivore diet based on NHANES 2017–2018 data (reference diet) and substituted animal products with either traditional plant-based foods or novel non-animal protein alternatives to create flexitarian, vegetarian and vegan diets matched for energy, protein, carbohydrate and fat, to focus on changes in micronutrient intake over a given day. The goal of these comparisons was to establish whether reducing the consumption of animal products using novel PBMA and dairy alternatives results in equivalent nutrient intakes for flexitarian, vegetarian and vegan diets, in comparison to a reference omnivore diet.

## 2. A Comparison of a Standard Omnivore Western Diet to Plant-Based Alternative Diets

### 2.1. Selecting a Representative Diet

We compared changes in nutrient intakes when moving from an animal-based diet (omnivore) to plant-based diets (flexitarian, vegetarian and vegan). For the comparison, we selected a reference diet based on the average nutrient intake pattern for an American adult male aged 20–49, based on the NHANES 2017–2018 dietary survey [36] (Table 1). This diet is within recommendations for daily calories and most micronutrients [22,36] but was higher for daily protein, carbohydrate, fat, sugar and sodium and lower in dietary fibre, potassium and magnesium (Table 2). This reference diet is representative of the typical intake of the average consumer since most individuals do not meet recommendations for all nutrients [37].

We compared the reference omnivore diet to traditional and newer versions of flexitarian diets with reduced meat intake (Flex-Trad and Flex-New), traditional and newer versions of a vegetarian diet with no meat (Veg-Trad and Veg-New), and traditional and newer versions of a vegan diet without meat, dairy or eggs (Vegan-Trad and Vegan-New) (Table 1). The Flex-Trad, Veg-Trad and Vegan-Trad diets substituted animal products in the reference diet for traditional plant-based substitutes such as beans, nuts and soy. For the Flex-New, Veg-New and Vegan-New diets, we substituted animal products for novel PBMAs and vegan-friendly packaged products such as coconut- or soy-based dairy alternatives, to represent the recent consumer trend towards these products. To focus comparisons on micronutrient intakes, all diets were matched to within 5% for total calories from carbohydrates, fat and protein (Table 2). Although these diets were hypothetical, they represent practical choices that are available within the current plant-based market and were chosen to be realistic, with nutrient values reflecting each food’s composition “as prepared” and ready-to-consume, rather than on-pack composition “as sold”. For detailed nutritional information for each individual food item in each diet, please refer to the Supplementary Materials (Tables S1–S7).

### 2.2. Summary Comparison of Reference Diet to Flexitarian, Vegetarian and Vegan Diets

**Figure 1:**
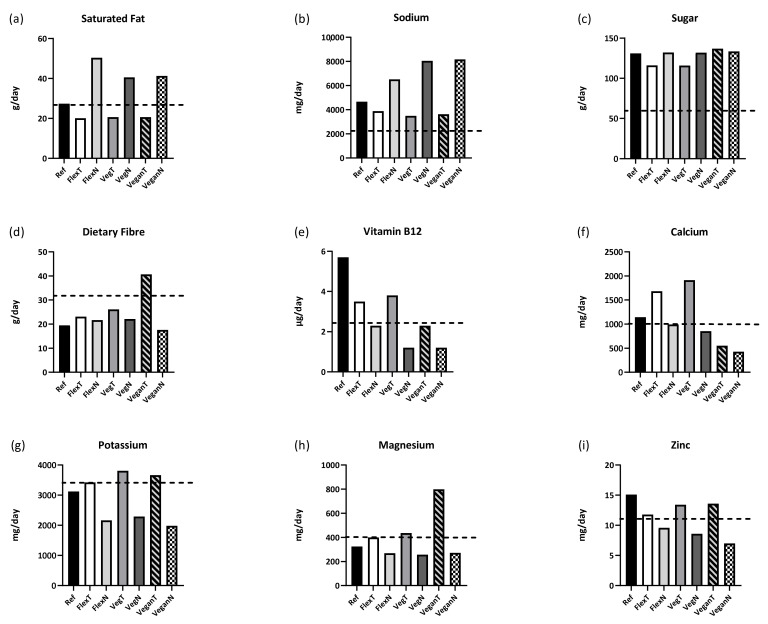
Comparison of nutrient intakes across omnivore, flexitarian (traditional and novel), vegetarian (traditional and novel) and vegan (traditional and novel) diets for (**a**) saturated fat, (**b**) sodium, (**c**) sugar, (**d**) dietary fibre, (**e**) Vitamin B12, (**f**) calcium, (**g**) potassium, (**h**) magnesium and (**i**) zinc. The dotted line in each graph denotes the daily nutritional goal for that nutrient [22]. Ref = reference diet, FlexT = flexitarian-traditional diet, FlexN= flexitarian-novel diet, VegT = vegetarian-traditional diet, VegN = vegetarian-novel diet, VeganT = vegan-traditional diet, VeganN = vegan-novel diet.

Flexitarian, vegetarian and vegan diets with less meat and dairy intakes were lower in cholesterol, Vitamin B12 and zinc (Table 2). All diets met daily requirements for phosphorus and iron but fell below fibre requirements (Table 2, Figure 1), with the exception of the traditional vegan diet, which had the greatest quantity of legumes and seeds. Diets with novel plant-based substitutes (Flex-New, Veg-New and Vegan-New) fell below daily requirements for calcium, potassium, magnesium, zinc and Vitamin B12 and exceeded the reference diet for saturated fat, sodium and sugar (Table 2, Figure 1). The shortfall in micronutrients was due to low or no meat and dairy consumption, and the fact that many novel PBMAs had lower micronutrient contents compared to equivalent animal products and many traditional plant-based foods. The increase in public health-sensitive nutrients was due to preparation methods requiring oil and salt and condiments consumed with PBMAs. Many vegan-friendly coconut-based products were low in protein and micronutrients and high in fat. Diets with traditional plant-based substitutes (Flex-Trad and Veg-Trad) apart from the vegan diet (Vegan-Trad) met daily requirements for calcium, potassium, magnesium, phosphorus, zinc, iron and Vitamin B12, and was lower than the reference diet in saturated fat, sodium and sugar (Table 2, Figure 1). This was due to lower meat and dairy intakes and an increase in legumes and seeds. Traditional flexitarian and vegetarian diets were the only two diets to meet all daily micronutrient requirements (Figure 1).

### 2.3. Comparison of the Reference Omnivore Diet to Two Alternative Flexitarian Diets

The flexitarian diets (Flex-Trad and Flex-New) had a reduced proportion of meat and dairy while increasing plant-based foods, and both were lower in cholesterol, Vitamin B12 and zinc and higher in fibre than the reference diet. The traditional flexitarian diet met daily requirements for all seven micronutrients compared and was lower in saturated fat (27%) and sodium (↓17%) and higher in calcium (↑32%) and magnesium (↑19%) than the reference diet. This was due to a higher proportion of low-fat dairy and plant-based foods such as legumes, which were important protein sources for this diet. The flexitarian diet based on more novel products (Flex-New) only met daily requirements for phosphorus and iron and was the diet highest overall for saturated fat. This was due to a greater proportion of novel plant-based convenience foods such as plant-based snacks, coconut-based products and lower dairy intake. Flex-New had 60% less Vitamin B12, 37% less zinc and 31% less potassium than the reference diet while increasing saturated fat intake by 46% and sodium by 28% (Figure 1, Table 2).

### 2.4. Comparison of the Reference Omnivore Diet to Two Alternative Vegetarian Diets

Removing meat entirely, while retaining dairy and increasing the proportion of energy derived from plant-based foods, resulted in traditional (Veg-Trad) and novel (Veg-New) vegetarian diets, both of which were lower than the reference diet for cholesterol, Vitamin B12 and zinc, and higher in dietary fibre and iron. As with the traditional flexitarian diet, the traditional vegetarian diet met daily requirements for all micronutrients compared and was lower in saturated fat (↓24%) and sodium (↓25%) and higher in calcium (↑40%) and magnesium (↑26%) than the reference diet. This was due to increased intake of dairy and plant-based products such as vegetables, beans, tofu and nuts. The novel vegetarian diet only met daily requirements for phosphorus and iron and not the other micronutrients, due to a greater quantity of novel PBMAs and coconut-based snack products. As a result, the novel vegetarian diet was markedly lower in Vitamin B12 (↓79%), zinc (↓43%), potassium (↓27%) and calcium (↓25%) compared to the reference diet. The novel vegetarian diet also exceeded the reference diet for saturated fat (↑32%) and sodium (↑42%).

### 2.5. Comparison of the Reference Omnivore Diet to Two Alternative Vegan Diets

Excluding all meat and dairy resulted in vegan diets with no cholesterol and lower Vitamin B12, calcium and zinc content compared to the reference diet. These vegan diets were also higher than the reference diet for iron and sugar. The traditional vegan diet had the highest quantity of legumes and seeds and was the diet highest for iron and fiber, and higher in potassium (↑15%) and magnesium (↑59%) compared to the reference diet. However, the traditional vegan diet also had less Vitamin B12 (↓59%) and calcium (↓52%) than the reference diet. Soymilk was the sole source of Vitamin B12 for both vegan diets. The traditional vegan diet (Vegan-Trad) met all daily micronutrient requirements with the exception of Vitamin B12 and calcium. The novel vegan diet (Vegan-New) provided adequate phosphorus and iron but was the diet lowest for calcium, potassium, phosphorus, zinc and Vitamin B12. Removing all dairy and meat products resulted in daily requirements not being met for Vitamin B12 (79% less than reference diet), calcium (↓62%), potassium (↓36%), magnesium (↓16%) and zinc (↓54%). The novel vegan diet was higher than the reference diet for saturated fat (↑34%) and sodium (↑43%) and had the lowest fibre of all the diets due to the greatest proportion of novel PBMAs and plant-based snack foods. Since many novel PBMAs were rich in iron due to the inclusion of vegetable haem or ferrous sulfate, the novel vegan diet had one of the highest overall iron contents.

## 3. Trading Places: Feasibility and Nutritional Balance within Specific Diet Comparisons

The traditional flexitarian diet reduced meat using traditional plant-based substitutes and met all daily micronutrient requirements. Retaining dairy and eggs with a smaller portion of meat maintained protein intakes, as well as sustained high micronutrient intakes. This diet did not require the addition of high-protein snacks to meet protein needs, as was needed for vegetarian and vegan diets. An added challenge when making the transition to vegetarian and vegan diets is that unlike meat, many plant-based protein sources tended to be high in carbohydrates, such as dairy and legumes. Similarly, plant sources such as tofu are often lower in protein compared to meats like chicken and need to be consumed in larger portions or supplemented with high-protein snacks such as nuts and yogurt to achieve sufficient intakes, which can increase dietary fat and carbohydrate. The novel vegetarian and vegan diets with PBMAs and dairy alternatives such as coconut ice cream and coconut yogurt were matched for protein intake with the other diets. PBMAs often had lower protein and higher fat, carbohydrate and sodium compared to meat, while dairy alternatives tended to have lower protein yet higher calories, carbohydrate and fat compared to traditional dairy. Non-dairy milk had more added sugar. Accommodating protein from PBMAs and dairy alternatives while matching for energy intake required a reduction in carbohydrate-rich foods such as French fries, rice and bread.

For vegans to go dairy-free, this required the removal of nutrient-rich foods such as cheese and replacing these with dairy-free cheese, which was higher in calories, carbohydrate, fat and sodium but lacked protein. Several foods used to replace animal-source foods in each diet were also low in micronutrients such as coconut yogurt, plant-based egg and dairy-free cheese. To match the reference diet for protein, the novel vegan diet required “supplementation” with protein-rich plant-based snacks such as vegan “beef jerky”. Significant protein sources in the vegan diets such as legumes, seeds and PBMAs increased carbohydrate and fat intakes. Whereas vegan diets are known for their low Vitamin B12 content [43,44], this can be overcome with specific vegan food choices such as yeast-fortified dairy-free cheese.

When modelling the various diets to be used in our comparisons, we were mindful of the high variability in nutritional characteristics among products such as plant-based drinks or dairy alternatives [45]. Therefore, to reduce the influence of variability, our case study excluded fortified products and supplements for an equivalent comparison across diets. As a result, the vegan diets had no dietary sources of Vitamin B12 other than soymilk, and neither vegan diet met daily requirements for all seven micronutrients compared, with Vegan-New being the lowest for five of these micronutrients. This suggests that consumption of these diets could result in important micronutrient deficiencies if sustained over time and that vegetarian and vegan diets may require more consideration and dietetic knowledge to implement if they are to meet all daily micronutrient requirements. This includes incorporating a greater proportion of traditional plant-based foods such as beans, nuts and seeds, rather than selecting nutrient-poor PBMAs, and selecting calcium-fortified dairy alternatives alongside Vitamin B12 supplements.

The reference diet against which all other diets were compared reflects a typical daily diet for an American adult male. When calories and % energy from macronutrients were matched, all diets exceeded requirements for macronutrients, sodium and sugar. Only the traditional flexitarian and vegetarian diets met all daily micronutrient requirements, with better intakes than the reference diet. The diets selected for comparison aimed to illustrate in a practical way the possible nutritional implications of adopting flexitarian, vegetarian or vegan diets for the long term. Often, when comparing animal- and plant-based diets, the focus tends to be exclusively on protein quality and quantity. While this is important, the current comparison highlights important implications of selecting plant-based foods believed to be “healthier”, and the need to consider non-animal alternatives that are nutrient-dense, and lower in calories, fat, sugar and sodium.

## 4. Discussion

The current case study demonstrates that traditional plant-based replacements for animal products support the transition to nutritionally adequate flexitarian or vegetarian diets, with flexitarian diets being the most feasible overall. Plant-based diets with larger proportions of novel PBMA substitutes and vegan diets in the absence of nutritional supplements run the risk of being inadequate in a number of important micronutrients.

### 4.1. Potential Unintended Consequences of Switching to a Plant-Based Diet

A diet that reduces or excludes meat and dairy may have unintended nutritional consequences that arise through selecting foods of lower nutrient density or foods requiring preparation with oil or salt. The current case study shows that diets that increasingly avoid animal-based products result in nutritionally adequate diets if traditional plant-based foods are used as substitutes, with the exception of vegan diets. However, we identified risks when the move to vegetarian and vegan diets was achieved by including novel PBMAs and plant-based dairy, with significantly decreased Vitamin B12, calcium, potassium, magnesium and zinc.

The current comparisons were based on a single day’s consumption, which suggests that the continued consumption of diets high in novel PBMAs and plant-based dairy carry the potential risk of increasing intakes of public health-sensitive nutrients while also promoting nutritional deficiencies for a range of micronutrients. For the uninformed consumer, caution should be exercised when making wholesale transitions from diets containing food of animal origin to plant-based diets, particularly those moving to a vegan diet. A diet with sustained low calcium content can increase the risk of low bone mineral density, osteoporosis and fractures. The vegan diets in our case study were the lowest in calcium and the Vegan-New diet was lowest in zinc. These findings are in agreement with a recent cross-sectional comparison that found poorer bone health in vegans compared to omnivores, as well as lower nutritional biomarkers including calcium and zinc [46]. Recent evidence has shown an acceleration of bone turnover when shifting from animal- to plant-based diets [47]. As such, those considering the move to veganism should take steps to ensure adequate consumption of all micronutrients including calcium, zinc and Vitamin B12. As mentioned earlier, this may include calcium-fortified dairy alternatives, Vitamin B12 supplements and traditional plant-based foods. Similarly, consideration should be given to limit specific vegan food choices high in fat, sodium and sugar. This may include several foods catered to vegans, for example falafel and halloumi, which are commonly fried, and non-dairy alternatives such as vegan cheese, yogurt and ice cream, which tend to be lower in protein and micronutrients when compared to animal-based dairy products.

Previous research using dietary data modelling has estimated the impact of switching from animal- to plant-based diets on nutrient intakes. A Canadian study found that increasing the intake of PBMAs by 100% and reducing red and processed meat by 50% improved diet quality as measured by the Nutrient-Rich Foods (NRF) index, but decreased intakes of important nutrients including protein, zinc and Vitamin B12 [48]. Similarly, a French diet simulation study substituted meat, milk and dairy desserts with plant-based substitutes, finding better adequacy for nutrients such as essential fatty acids and fibre but lower adequacies for Vitamin B12, riboflavin, zinc, iron and calcium [49]. There remains a shortage of long-term evidence on the health effects of substituting animal-based foods for newer plant-based meat, dairy and eggs. Two randomized controlled trials to date suggest either a potential long-term negative impact on bone health [47] or beneficial effects including reduced low-density lipoprotein (LDL) cholesterol in an industry-funded trial [50]. However, long-term trials have yet to be completed, and the fluidity of PBMA product formulations and diverse range of products present additional challenges to interpreting the impact of consumption data on health in the future.

Findings from the current case study align with existing literature that flexitarian diets may be the easiest for consumers to transition to in terms of nutritional adequacy and improved health, whereas nutritionally adequate vegetarian and vegan diets require more knowledge and planning. Consuming a mix of proteins from animals and plants while reducing meat and increasing healthful plants is a practical approach for many [51,52]. However, the nutritional profiles of these flexitarian diets depend on the dietary components used as substitutes for animal products [51]. Similarly, when sustaining an omnivorous diet, it is possible to achieve nutrient balance when meat and dairy are low in saturated fat [53] and nutrient-dense foods are prepared with less salt, sugar and oil. However, evidence suggests that reducing meat consumption can have additional health benefits over a balanced omnivore diet. A recent review of Canadian epidemiological studies supports consumption of well-planned vegetarian or vegan diets over omnivore diets to improve nutritional adequacy and reduce risks of chronic conditions and cancer [54]. The current case study suggests consumers will need support and guidance to ensure that when they reduce consumption of animal products, they choose alternatives that avoid unintentional health effects of sustained consumption of nutrient-poor plant-based products.

### 4.2. Making the Switch from Animals to Plants: Nutrient Density versus Protein Quantity and Quality

Much of the commentary around shifting from animal- to plant-based protein diets has centered on the poor protein quantity and quality of plant-based foods [4,30]. However, our comparisons highlight that protein quality or quantity is unlikely to be an issue and many plant proteins and protein blends are capable of meeting daily protein requirements, particularly when consuming a variety of plant foods. Concerns have been raised about consuming adequate protein from vegetarian and vegan diets [14,15], yet today a majority of consumers in developed countries tend to exceed protein requirements [36]. The current comparison suggests the challenge for the emerging plant-based product market will be to enhance nutrient-poor plant sources with adequate micronutrients while reducing consumers’ need to add public health-sensitive nutrients such as sugar, salt and fat to enhance the palatability of these products. The protein in new PBMAs is largely based on soy and pea protein and generally sufficient in terms of quantity and quality [50], albeit lower in protein content compared to their animal counterparts. However, the lower nutrient content and requirement for many products to be fried in oil and seasoned with salt could be problematic if adopted as a dietary staple, and the long-term impact of consuming novel PBMAs as a direct replacement for animal-based protein has yet to be tested. In this regard, switching to more frequent PBMA consumption may have the unintended consequence of increasing intakes of nutrient-poor “fast foods”, which in turn may not impact protein adequacy but could promote higher intakes of fat, sugar, salt and energy, thus reducing the micronutrient quality of the diet, as highlighted in Table 2 and Figure 1. In recognition of this, recently, PBMA producers such as Beyond Meat have started to fortify their products with B vitamins in order to create burger patties with a micronutrient profile more comparable to that of beef [55].

### 4.3. Long-Term Impact of Increasing Intakes of Novel PBMAs

Alongside the rapid rise of PBMAs, meat consumption has continued to increase. Global per capita meat consumption has increased approximately 20 kg since 1961 [56], raising the question of whether PBMAs are replacing meat or merely contributing to the increase in overall protein and energy intakes. The long-term health effects of increased PBMA intakes also remain unknown. Excessive haem consumption, the form of iron found in meat, has been associated with increased risks of cancer and type 2 diabetes [57,58,59,60,61,62,63]. These associations have not been found for non-haem iron found in plants, which has been used in novel PBMA products to impart the qualities of minced beef. Reducing meat and increasing vegetable intake in an iron-sufficient diet carries health benefits such as promoting the prevention of cancer and chronic diseases [64]. It is unknown whether novel, processed PBMAs carry these same benefits, for instance, soy leghaemoglobin in Impossible Foods. Extensive research over many years has demonstrated the benefits of reducing meat and adopting traditional flexitarian, vegetarian and vegan diets, with associated reductions in cardiovascular disease and cancer risk [17,19]. Claims of the health benefits of novel PBMAs are currently not supported by the same robust long-term evidence from randomised controlled trials, yet these “health halos” are contributing to consumer motivations to consume PBMAs in an effort to improve their health. As highlighted earlier, this may unintentionally support a move to more unbalanced diets and increase intakes of unhealthy dietary components. In the past, unsubstantiated health claims have been shown to drive consumer choice and intake behaviour. For example, when foods are labelled “organic”, consumers perceive them to be healthier and to contain fewer calories, despite no evidence to support this [65,66,67]. Research is now needed to understand the health implications of consuming PBMA products and identify opportunities to enhance their health profile.

Novel PBMAs are marketed to appeal to consumers looking to adopt a “clean” eating dietary regime as part of a global trend to simplify food production and formulations in favour of more natural food products [68]. However, this desire for clean eating conflicts with many novel PBMAs which are highly formulated, processed products that rely on protein isolates, colours, flavours and processing aids to achieve a “meat-like” sensory appeal. A recent study found a greater proportion of “ultra-processed foods” (UPFs) in diets that avoided animal-based foods, with UPFs supplying 33% of daily calories for meat eaters and 39.5% for vegans [69]. Another study saw substitutions of meat, milk and dairy desserts for plant-based substitutes to modify the energy share of UPFs from 29% to 27–40% depending on individual foods used [49]. Thus, many consumers seeking to avoid processed foods when shifting to plant-based diets, especially vegan diets, may find themselves conflicted when faced with messages to consume more of the novel PBMAs. Though processed foods have been associated with increased disease risk [70], not all are unhealthful (i.e., tofu and fortified foods), and it is not clear whether aspects of processing or food formulation are primarily associated with diet-related chronic disease. There is potential for food technology to develop affordable, sustainable and nutritious meat and milk analogues to enhance the nutritional content of diets and benefit consumer health [71]. Our case study comparisons identified a need to enhance the nutrient densities of plant-based proteins, especially novel versions, to create products higher in bioavailable nutrients and lower in public-health-sensitive nutrients. Sugar can be reduced in dairy alternatives while increasing amounts of protein, calcium, Vitamin B12 and other nutrients less often found in non-dairy products. In addition, rather than attempting to construct foods replicating meat or dairy, it may be more nutritious and feasible to encourage consumers to reduce meat and increase consumption of existing nutrient-dense, natural plant sources. Investments and marketing could focus on promoting intakes of fresh vegetables, legumes and seeds rich in protein instead of PBMA products, which are not equivalent nutritionally (as highlighted in Section 2) and are more costly for consumers [9]. Improving the availability of fruits and vegetables in schools, workplaces and communities coupled with choice architecture and strategies to increase palatability of such foods have shown promise [72].

Rather than classifying foods by the degree to which they are processed to determine their healthfulness, measures have been developed that better reflect not only the nutrient density but also the sustainability of foods, which could be used to better inform consumers. Measures such as the plant-based diet index (PDI) may better reflect the nutrient density and impact of dietary changes on health [73]. High-quality plant foods such as wholegrains, fruits, vegetables, nuts and legumes are scored high on the healthful plant-based diet index (hPDI), and lower-quality plant foods such as those high in added sugars and refined grains are high on the unhealthful plant-based diet index (uPDI). In large prospective cohorts, the hPDI was more strongly associated with decreased coronary heart disease (CHD) risk and the uPDI associated with increased CHD risk [73]. Drewnowski and colleagues examined the association between greenhouse gas emissions and energy and nutrient densities of 34 food categories [74]. These approaches could be used to create a standardised diet index that accounts for both the nutritional value and environmental impact of foods selected and consumed. This could be applied to better inform consumers by helping them identify nutrient-dense alternatives to animal products, thus assisting the healthful transition towards sustainable plant-based diets.

This case study’s strength was the modelling of seven diets (omnivore, flexitarian, vegetarian and vegan) in a practical manner that follows current nutrient intakes to represent the average consumer. However, it had its limitations. We did not include supplements or fortified products in order to create an equivalent comparison across diets. Therefore, this case study will not fully represent the diets of plant-based consumers who use supplements and fortified products. However, cross-sectional and survey data suggest that supplement use may be lower than expected [43,44] despite an increased risk of poorer micronutrient density in plant-based diets such as vegan diets. Additionally, the single-day dietary pattern, while allowing us to go into detailed comparisons in our data for seven unique diets (i.e., serving sizes, calories and 15 nutrient values of individual foods), forms another limitation of the case study.

## 5. Conclusions

The current case study highlights that traditional plant-based substitutes can improve nutrient intakes and help consumers to eat more sustainably. However, our findings suggest that it is easy for the uninformed consumer to unintentionally increase public health-sensitive nutrients such as fat, sodium and sugar while also decreasing the nutrient density of the diet. Recent innovation in the plant-based product space has focused more on organoleptic properties (texture, taste and appearance) and formats (nuggets and burgers), rather than developing innovative ways to enhance the nutrient density of plant-derived foods, and ensuring a balanced nutrient profile similar to products of animal origin. Many newer plant-based products are similar to animal products in calories, but lower in protein, calcium, potassium, magnesium, zinc and Vitamin B12 while being higher in sodium and fat after being prepared. If habitually consumed, this could create nutrient shortfalls for consumers motivated to follow healthier, more sustainable diets [8].

Research is needed on how best to guide consumers to choose nutrient-dense plant-based diets that support reduced consumption of public health-sensitive nutrients while sustaining protein quantity and quality. For food producers, there is potential to innovate for the next generation of plant-based foods that provide adequate nutrient intakes alongside protein, and for the development of product formats that do not require the addition of salt, sugar and fat to enhance their sensory appeal.

## Figures and Tables

**Table 1:** Foods Used for Reference, Flexitarian, Vegetarian and Vegan Diets.

	Reference Diet	Flexitarian-Traditional (Flex-Trad)	Flexitarian-Novel (Flex-New)	Vegetarian-Traditional (Veg-Trad)	Vegetarian-Novel (Veg-New)	Vegan-Traditional (Vegan-Trad)	Vegan-Novel (Vegan-New)
	Example of a Typical Western Diet	Less Meat Traditional Plant Sources	Less Meat Novel Plant Sources	No Meat Traditional Plant Sources	No Meat Novel Plant Sources	No Meat or Dairy Traditional Plant Sources	No Meat or Dairy Novel Plant Sources
**Breakfast**	White bread (2 slices, 95 g) Butter (13 g) Bacon (29 g) Hardboiled egg (38 g) Low-fat milk (200 mL)	White bread (2 slices, 95 g) Olive-based margarine (14 g) Egg omelette with spinach and low-fat cheese (129 g) Low-fat milk (200 mL)	White bread (2 slices, 95 g) Olive-based margarine (7 g) Egg omelette (85 g) Bacon, meatless (16 g) Ketchup (8 g) Low-fat milk (200 mL)	White bread (2 slices, 95 g) Olive-based margarine (18 g) Egg omelette with spinach and low-fat cheese (119 g) Low-fat milk (200 mL)	White bread (2 slices, 95 g) Olive-based margarine (7 g) Plant-based egg omelette with dairy-free cheese (115 g) Bacon, meatless (18 g) Ketchup (15 g) Low-fat milk (200 mL)	White bread (1 slice, 48 g) Peanut butter (15 g) Plant-based egg omelette with black beans (139 g) Soymilk (300 mL)	White bread (2 slices, 95 g) Coconut spread (14 g) Plant-based egg omelette (85 g) Bacon, meatless (43 g) Ketchup (15 g) Soymilk (300 mL)
**Morning Snack**	Black coffee (250 mL)	Black coffee (250 mL)	Black coffee (250 mL) Plant-based beef jerky (38 g)	Strawberries (100 g) Black coffee (250 mL)	Black coffee (250 mL) Plant-based beef jerky (35 g)	Strawberries (100 g) Black coffee (250 mL)	Plant-based beef jerky (30 g) Black coffee (250 mL)
**Lunch**	Beef burger with low-fat cheese (245 g) French fries (117 g) Soda (620 mL)	Bean burrito with low-fat cheese (281 g) French fries (117 g) Soda (620 mL)	Plant-based burger with low-fat cheese (257 g) French fries (31 g) Soda (620 mL)	Bean burrito with low-fat cheese (281 g) French fries (117 g) Soda (620 mL)	Plant-based burger with low-fat cheese (252 g) French fries (71 g) Soda (620 mL)	Bean burrito with hummus and dairy-free cheese (266 g) French fries (31 g) Soda (620 mL)	Plant-based burger with dairy-free cheese (268 g) French fries (31 g) Soda (620 mL)
**Afternoon Snack**	Low-fat yogurt (140 g) Strawberries (100 g)	Unsalted nuts (35 g) Peach (150 g)	Strawberries (100 g) Coconut yogurt (170 g)	Unsalted nuts (25 g) Peach (150 g)	Strawberries (100 g) Plant-based beef jerky (35 g)	Pumpkin seeds (30 g) Soymilk (300 mL)	Plant-based beef jerky (30 g) Unsalted nuts (8 g)
**Evening Meal**	Grilled chicken (100 g) Stir-fried spinach (91 g) White rice (125 g)	Grilled chicken (100 g) Stir-fried broccoli (102 g) White rice (85 g)	Plant-based chicken tenders (104 g) Ketchup (8 g) Stir-fried broccoli (93 g) White rice (100 g)	Tofu (127 g) Stir-fried broccoli and spinach (115 g) White rice (75 g)	Plant-based chicken tenders (104 g) Ketchup (15 g) Stir-fried broccoli (93 g) White rice (60 g)	Tofu (157 g) Black beans (81 g) Stir-fried broccoli (94 g) White rice (25 g)	Plant-based chicken tenders (104 g) Ketchup (15 g) Stir-fried broccoli (97 g) White rice (25 g)
**Dessert**	Ice cream (80 g) Peach (150 g)	Low-fat yogurt (125 g) Banana (50 g)	Vegan coconut ice cream (55 g) Peach (150 g)	Low-fat yogurt (220 g) Unsalted nuts (25 g)	Vegan coconut ice cream (80 g) Peach (150 g)	Coconut yogurt (50 g) Peach (150 g) Pumpkin seeds (30 g)	Strawberries (100 g) Peach (150 g)
**Total**	2482 kcal, 290 g carbohydrate, 105 g protein, 102 g total fat	2478 kcal, 304 g carbohydrate, 103 g protein, 97 g total fat	2435 kcal, 277 g carbohydrate, 99 g protein, 106 g total fat	2486 kcal, 304 g carbohydrate, 101 g protein, 100 g total fat	2517 kcal, 297 g carbohydrate, 108 g protein, 106 g total fat	2514 kcal, 304 g carbohydrate, 99 g protein, 107 g total fat	2471 kcal, 291 g carbohydrate, 104 g protein, 105 g total fat

Values for mixed foods such as omelettes, burgers, burritos and stir-fried vegetables differ across diets due to varying amounts of individual ingredients such as oil, salt and cheese. Nutrient values for each food item are taken from the United States Department of Agriculture [38]; Health Promotion Board, Singapore [39]; and Internet sources [11,40,41,42]. These diets used unfortified products and assumed no multivitamins nor supplements were taken.

**Table 2:** Nutritional Compositions of Reference, Flexitarian, Vegetarian and Vegan Diets.

	Calories (kcal)	Carbs (g)	Protein (g)	Total Fat (g)	Sat Fat (g)	Chol (mg)	Sodium (mg)	Sugar (g)	Fibre (g)	B12 (ug)	Calcium (mg)	Potassium (mg)	Magnesium (mg)	Phosphorus (mg)	Zinc (mg)	Iron (mg)
**Reference**	2482.1	290.4	104.5	101.9	27.4	383.4	4663.5	130.9	19.5	5.7	1143.0	3122.8	324.5	1589.1	15.1	16.0
**Flex-Trad**	2478.0	304.0	103.1	96.9	20.1	309.3	3882.3	116.0	23.1	3.5	1685.1	3424.8	400.7	1946.4	11.8	15.1
**Flex-New**	2434.8	276.6	99.4	106.1	50.4	325.2	6514.0	132.2	21.7	2.3	992.7	2169.8	269.4	1586.3	9.6	20.4
**Veg-Trad**	2486.1	304.0	100.9	100.3	20.7	235.2	3497.8	115.8	26.1	3.8	1913.4	3808.3	435.5	2091.2	13.4	16.8
**Veg-New**	2516.7	296.8	107.8	105.8	40.5	23.4	8053.0	131.8	22.1	1.2	853.9	2291.5	256.9	1545.6	8.6	19.3
**Vegan-Trad**	2514.1	303.9	99.3	107.0	20.7	0	3621.8	136.9	40.7	2.3	552.8	3666.3	799.7	2068.6	13.6	22.2
**Vegan-New**	2471.2	290.7	104.4	105.4	41.3	0	8166.4	133.4	17.6	1.2	429.6	1987.9	272.1	1308.5	7.0	20.4
**Dietary Guidelines**	2200–2400	130	56	20–35% (49–93 g)	<27	-	2300	<60	31	2.4	1000	3400	400	700	11	8

Dashes denote unavailable values. Carbs = carbohydrate; Sat Fat = saturated fat; Chol = cholesterol; Fibre = Dietary Fibre; B12 = Vitamin B12. Nutrient values for each food item are taken from the United States Department of Agriculture [38]; Health Promotion Board, Singapore [39]; and Internet sources [11,40,41,42]. Dietary guidelines are the 2020–2025 Dietary Guidelines for Americans [22].

## Data Availability

Data presented are from publicly available online databases: the United States Department of Agriculture’s FoodData Central database [36] and Singapore’s Health Promotion Board’s Energy & Nutrient Composition of Food database [37].

## Supplementary Materials

The following are available online at https://www.mdpi.com/article/10.3390/nu13082527/s1, Table S1: Reference Diet; Table S2: Flexitarian-Traditional (Flex-Trad) Diet; Table S3: Flexitarian-Novel (Flex-New) Diet; Table S4: Vegetarian-Traditional (Veg-Trad) Diet; Table S5: Vegetarian-Novel (Veg-New) Diet; Table S6: Vegan-Traditional (Vegan-Trad) Diet; Table S7: Vegan-Novel (Vegan-New) Diet.
