# Supplementary material for: Unintended Consequences: Nutritional Impact and Potential Pitfalls of Switching from Animal- to Plant-Based Foods
Source: Nutrients. 2021 Jul 23;13(8):2527. doi: 10.3390/nu13082527 (PMC8398225; doi:10.3390/nu13082527)
Supplement: Supplementary file 1 [file nutrients-13-02527-s001.zip › nutrients-1300073-supplementary.pdf]

Supplementary Material

Table S1: Reference Diet

	Item	Serving	Calories	Carbs (g)	Protein (g)	Total Fat (g)	Sat Fat (g)	Chol (mg)	Sodium (mg)	Sugar (g)	Dietary Fibre (g)	B12 (ug)	Calcium (mg)	Potassium (mg)	Magnesium (mg)	Phosphorus (mg)	Zinc (mg)	Iron (mg)
**Breakfast**	White bread[1]	2 slices, 95g	237.50	47.50	6.48	2.16	0	0	453.41	4.32	2.18	0	86.36	111.15	34.11	97.85	0.84	4.64
	Butter[1]	13g	93.38	0.008	0	10.53	6.67	27.92	83.58	0.008	0	0.02	3.12	3.12	0.26	3.12	0.01	0.003
	Bacon[1]	29g	134.30	0.17	2.64	13.84	1.11	6.24	873.63	0.12	0	0.05	1.26	26.29	2.62	31.20	0.16	0.06
	Hardboiled egg[1]	38g	58.90	0.43	4.78	4.03	1.24	141.36	47.12	0.43	0	0.42	19.00	47.88	3.80	65.36	0.40	0.45
	Low fat milk[1]	200ml	80.00	9.10	6.34	2.02	1.27	8.00	102.00	9.10	0	0.70	226.00	314.00	22.00	170.00	0.74	0.06
**Morning Snack**	Black coffee[1]	250ml	5.00	0.85	0.25	0	0.01	0	10.00	0	0	0	10.00	75.00	10.00	7.50	0.03	0.10
**Lunch**	Beef burger with low fat cheese[1]	245g	487.60	28.18	40.05	23.53	7.79	100.65	932.65	12.26	6.91	3.17	305.65	504.27	61.35	425.65	7.89	3.05
	French fries[1,2]	117g	365.00	48.40	4.01	17.20	2.74	0	246.00	0	4.00	0	21.10	527.67	43.30	146.00	0.59	0.95
	Soda[1,2]	620ml	210.00	56.00	0	0	0	0	55.00	56.00	0	0	6.20	31.00	0	55.80	0.56	0.12
**Afternoon Snack**	Low fat yogurt[1]	140g	88.12	9.88	7.35	2.17	1.40	8.40	98.00	9.88	0	0.78	256.12	327.76	23.80	201.76	1.24	0.11
	Strawberries[1]	100g	32.00	7.68	0.67	0.30	0.02	0	1.00	4.89	2.00	0	16.00	153.00	13.00	24.00	0.14	0.41
**Evening Meal**	Grilled chicken[1]	100g	202.35	7.34	21.18	9.15	2.11	78.00	454.12	5.98	0.20	0.14	12.00	280.00	22.00	160.00	0.74	0.55
	Stir-fried spinach[1,2]	91g	150.85	5.88	2.29	13.76	1.03	0	906.05	0.27	1.51	0	64.88	395.78	51.62	42.95	0.40	1.85
	White rice[1]	125g	161.25	34.99	3.34	0.35	0.10	0	306.25	0.06	0.50	0	12.50	43.75	15.00	53.75	0.61	1.49
**Dessert**	Ice cream[1,3]	80g	112.80	18.75	3.75	2.50	1.87	12.80	75.20	15.00	0	0.40	96.80	99.20	9.60	71.20	0.42	1.65
	Peach[1]	150g	63.00	15.20	1.36	0.41	0.03	0	19.50	12.60	2.25	0	6.00	183.00	12.00	33.00	0.35	0.51
**Total**			**2482.1**	**290.4**	**104.5**	**101.9**	**27.4**	**383.4**	**4663.5**	**130.9**	**19.5**	**5.7**	**1143.0**	**3122.8**	**324.5**	**1589.1**	**15.1**	**16.0**

Sources: [1] United States Department of Agriculture [1], [2] Internet sources [2], [3] Health Promotion Board [3]. Nutritional information of all food items (as prepared and ready-to-eat) was calculated by adding fat (oil), and sodium (salt) and/or garnishes such as garlic to raw ingredients. Dashes denote values not listed on nutritional labels or in databases. Carbs = carbohydrate; Sat Fat = saturated fat; Chol = cholesterol; B12 = Vitamin B12.

Table S2: Flexitarian-Traditional (Flex-Trad) Diet

	Item	Serving	Calories	Carbs (g)	Protein (g)	Total Fat (g)	Sat Fat (g)	Chol (mg)	Sodium (mg)	Sugar (g)	Dietary Fibre (g)	B12 (ug)	Calcium (mg)	Potassium (mg)	Magnesium (mg)	Phosphorus (mg)	Zinc (mg)	Iron (mg)
**Breakfast**	White bread[1]	2 slices, 95g	237.50	47.50	6.48	2.16	0	0	453.41	4.32	2.18	0	86.36	111.15	34.11	97.85	0.84	4.64
	Olive-based margarine[1,2]	14g	70.28	0.07	0.04	7.90	1.54	0	37.38	0	0	0.003	0	2.38	0.14	0.42	0	0
	Egg omelette with spinach and low fat cheese[1]	129g	267.05	2.84	17.09	20.70	3.79	198.25	155.35	1.15	0.66	1.14	391.05	269.25	41.30	312.45	2.05	1.47
	Low fat milk[1]	200ml	80.00	9.10	6.34	2.02	1.27	8.00	102.00	9.10	0.00	0.70	226.00	314.00	22.00	170.00	0.74	0.06
**Morning Snack**	Black coffee[1]	250ml	5.00	0.85	0.25	0	0.01	0	10.00	0	0	0	10.00	75.00	10.00	7.50	0.03	0.10
**Lunch**	Bean burrito with low fat cheese[1]	281g	415.60	58.22	25.70	8.33	3.75	17.50	1052.90	8.73	6.96	0.84	626.65	611.80	87.70	542.35	2.93	4.17
	French fries[1,3]	117g	365.00	48.40	4.01	17.20	2.74	0	246.00	0	4.00	0	21.10	527.67	43.30	146.00	0.59	0.95
	Soda[1,3]	620ml	210.00	56.00	0	0	0	0	55.00	56.00	0	0	6.20	31.00	0	55.80	0.56	0.12
**Afternoon Snack**	Unsalted nuts[1]	35g	205.45	7.44	8.52	17.38	2.70	0	2.10	1.72	2.94	0	20.30	221.90	62.30	127.05	0.97	0.55
	Peach[1]	150g	63.00	15.20	1.36	0.41	0.03	0	19.50	12.60	2.25	0	6.00	183.00	12.00	33.00	0.35	0.51
**Evening Meal**	Grilled chicken[1]	100g	202.35	7.34	21.18	9.15	2.11	78.00	454.12	5.98	0.20	0.14	12.00	280.00	22.00	160.00	0.74	0.55
	Stir-fried broccoli[1,2]	102g	123.90	9.06	2.78	9.31	0.76	0	998.30	1.42	2.24	0	39.80	296.30	17.88	66.30	0.40	0.70
	White rice[1]	85g	109.65	23.79	2.27	0.24	0.07	0	208.25	0.04	0.34	0	8.50	29.75	10.20	36.55	0.42	1.01
**Dessert**	Low fat yogurt[1]	125g	78.68	8.82	6.56	1.94	1.25	7.50	87.50	8.82	0	0.70	228.68	292.65	21.25	180.15	1.11	0.10
	Banana[1,2]	50g	44.50	9.40	0.55	0.17	0.06	0.00	0.50	6.10	1	0.00	2.50	179.00	16.50	11.00	0.08	0.13
**Total**			**2478.0**	**304.0**	**103.1**	**96.9**	**20.1**	**309.3**	**3882.3**	**116.0**	**23.1**	**3.5**	**1685.1**	**3424.8**	**400.7**	**1946.4**	**11.8**	**15.1**

Sources: [1] United States Department of Agriculture [1], [2] Health Promotion Board [3], [3] Internet sources [2]. Nutritional information of all food items (as prepared and ready-to-eat) was calculated by adding fat (oil), and sodium (salt) and/or garnishes such as garlic to raw ingredients. Dashes denote values not listed on nutritional labels or in databases. Carbs = carbohydrate; Sat Fat = saturated fat; Chol = cholesterol; B12 = Vitamin B12.

Table S3: Flexitarian-Novel (Flex-New) Diet

	Item	Serving	Calories	Carbs (g)	Protein (g)	Total Fat (g)	Sat Fat (g)	Chol (mg)	Sodium (mg)	Sugar (g)	Dietary Fibre (g)	B12 (ug)	Calcium (mg)	Potassium (mg)	Magnesium (mg)	Phosphorus (mg)	Zinc (mg)	Iron (mg)
**Breakfast**	White bread[1]	2 slices, 95g	237.50	47.50	6.48	2.16	0	0	453.41	4.32	2.18	0	86.36	111.15	34.11	97.85	0.84	4.64
	Olive-based margarine[1,2]	7g	35.14	0.04	0.02	3.95	0.77	0	18.69	0	0	0.001	0	1.19	0.07	0.21	0	0
	Egg omelette[1]	85g	162.32	0.93	10.06	13.04	6.40	297.60	875.20	0.90	0	0.89	40.53	100.96	8.02	137.60	0.84	0.96
	Bacon, meatless[1]	16g	59.33	0.71	1.52	6.11	2.50	0	383.80	0	0.34	0	3.13	22.14	2.48	9.10	0.06	0.32
	Ketchup[1]	8g	7.58	2.06	0.08	0.01	0	0	68.03	1.60	0.02	0	1.13	21.08	0.98	1.95	0.01	0.03
	Low fat milk[1]	200ml	80.00	9.10	6.34	2.02	1.27	8.00	102.00	9.10	0	0.70	226.00	314.00	22.00	170.00	0.74	0.06
**Morning Snack**	Black coffee[1]	250ml	5.00	0.85	0.25	0	0.01	0	10.00	0	0	0	10.00	75.00	10.00	7.50	0.03	0.10
	Plant-based beef jerky[1]	38g	121.98	10.86	13.57	1.36	0	0	868.68	5.43	0	0	0	0	7.30	145.01	0.75	1.63
**Lunch**	Plant-based burger with low fat cheese[1,3]	257g	506.07	31.01	36.99	26.70	9.61	19.60	1794.51	10.82	8.59	0.71	528.68	449.89	37.52	335.35	2.37	4.17
	French fries[1,3]	31g	96.71	12.82	1.06	4.56	0.73	0	65.18	0	1.06	0	5.59	139.81	11.47	38.68	0.16	0.25
	Soda[1,3]	620ml	210.00	56.00	0	0	0	0	55.00	56.00	0	0	6.20	31.00	0	55.80	0.56	0.12
**Afternoon Snack**	Strawberries[1]	100g	32.00	7.68	0.67	0.30	0.02	0	1.00	4.89	2.00	0	16.00	153.00	13.00	24.00	0.14	0.41
	Coconut yogurt[1]	170g	261.80	13.28	0	19.92	17.27	0	34.00	13.28	1.36	0	0	95.20	42.5	51	0.68	2.04
**Evening Meal**	Plant-based chicken tenders[1,3]	104g	193.13	15.70	13.73	9.83	1.65	0	1226.98	0	0	0	0.50	0.16	17.02	335.00	0.70	2.36
	Ketchup[1]	8g	7.58	2.06	0.08	0.01	0	0	68.03	1.60	0.02	0	1.13	21.08	0.98	1.95	0.01	0.03
	Stir-fried broccoli[1,2]	93g	65.56	9.08	2.78	2.89	2.24	0	222.30	1.42	2.24	0	39.35	296.14	17.86	66.30	0.40	0.69
	White rice[1]	100g	129.00	27.99	2.67	0.28	0.08	0	245.00	0.05	0.40	0	10.00	35.00	12.00	43.00	0.49	1.19
**Dessert**	Vegan coconut ice cream[1]	55g	161.15	13.75	1.79	12.56	7.77	0	2.75	10.16	1.21	0	12.10	120.04	20.08	33.00	0.45	0.86
	Peach[1]	150g	63.00	15.20	1.36	0.41	0.03	0	19.50	12.60	2.25	0	6.00	183.00	12.00	33.00	0.35	0.51
**Total**			**2434.8**	**276.6**	**99.4**	**106.1**	**50.4**	**325.2**	**6514.0**	**132.2**	**21.7**	**2.3**	**992.7**	**2169.8**	**269.4**	**1586.3**	**9.6**	**20.4**

Sources: [1] United States Department of Agriculture [1], [2] Health Promotion Board [3], [3] Internet sources [2,4,5], [4] On-pack nutritional labels. Nutritional information of all food items (as prepared and ready-to-eat) was calculated by adding fat (oil), and sodium (salt) and/or garnishes such as garlic to raw ingredients. Dashes denote values not listed on nutritional labels or in databases. Carbs = carbohydrate; Sat Fat = saturated fat; Chol = cholesterol; B12 = Vitamin B12.

Table S4: Vegetarian-Traditional (Veg-Trad) Diet

	Item	Serving	Calories	Carbs (g)	Protein (g)	Total Fat (g)	Sat Fat (g)	Chol (mg)	Sodium (mg)	Sugar (g)	Dietary Fibre (g)	B12 (ug)	Calcium (mg)	Potassium (mg)	Magnesium (mg)	Phosphorus (mg)	Zinc (mg)	Iron (mg)
**Breakfast**	White bread[1]	2 slices, 95g	237.50	47.50	6.48	2.16	0	0	453.41	4.32	2.18	0	86.36	111.15	34.11	97.85	0.84	4.64
	Olive-based margarine[1,2]	18g	90.36	0.09	0.05	10.15	1.98	0	48.06	0	0	0.004	0	3.06	0.18	0.54	0	0
	Egg omelette with spinach and low fat cheese[1]	119g	218.10	2.67	15.67	15.95	3.30	196.50	145.40	1.09	0.66	1.06	343.00	263.70	39.50	282.20	1.85	1.46
	Low fat milk[1]	200ml	80.00	9.10	6.34	2.02	1.27	8.00	102.00	9.10	0	0.70	226.00	314.00	22.00	170.00	0.74	0.06
**Morning Snack**	Strawberries[1]	100g	32.00	7.68	0.67	0.30	0.02	0	1.00	4.89	2.00	0	16.00	153.00	13.00	24.00	0.14	0.41
	Black coffee[1]	250ml	5.00	0.85	0.25	0	0.01	0	10.00	0	0	0	10.00	75.00	10.00	7.50	0.03	0.10
**Lunch**	Bean burrito with low fat cheese[1]	281g	415.60	58.22	25.70	8.33	3.75	17.50	1052.90	8.73	6.96	0.84	626.65	611.80	70.70	542.35	2.93	4.17
	French fries[1,3]	117g	365.00	48.40	4.01	17.20	2.74	0	246.00	0	4.00	0	21.10	527.67	43.30	146.00	0.59	0.95
	Soda[1,3]	620ml	210.00	56.00	0	0	0	0	55.00	56.00	0	0	6.20	31.00	0	55.80	0.56	0.12
**Afternoon Snack**	Unsalted nuts[1]	25g	146.75	5.32	6.09	12.42	1.93	0	1.50	1.23	2.10	0	14.50	158.50	44.50	90.75	0.69	0.40
	Peach[1]	150g	63.00	15.20	1.36	0.41	0.03	0	19.50	12.60	2.25	0	6.00	183.00	12.00	33.00	0.35	0.51
**Evening Meal**	Tofu[1,2]	127g	161.20	3.04	12.16	10.57	1.09	0.00	798.80	0.00	1.56	0.00	91.68	379.36	32.42	142.80	1.32	1.65
	Stir-fried broccoli and spinach[1,2]	115g	79.66	8.11	2.54	4.78	0.41	0.00	224.98	1.11	1.96	0.00	41.40	297.30	22.94	58.38	0.37	0.86
	White rice[1]	75g	96.75	20.99	2.00	0.21	0.06	0.00	183.75	0.04	0.30	0.00	7.50	26.25	9.00	32.25	0.37	0.89
**Dessert**	Low fat yogurt[1]	220g	138.47	15.53	11.54	3.42	2.20	13.20	154.00	15.53	0.00	1.23	402.47	515.06	37.40	317.06	1.95	0.18
	Unsalted nuts[1]	25g	146.75	5.32	6.09	12.42	1.93	0	1.50	1.23	2.10	0	14.50	158.50	44.50	90.75	0.69	0.40
**Total**			**2486.1**	**304.0**	**100.9**	**100.3**	**20.7**	**235.2**	**3497.8**	**115.8**	**26.1**	**3.8**	**1913.4**	**3808.3**	**435.5**	**2091.2**	**13.4**	**16.8**

Sources: [1] United States Department of Agriculture [1], [2] Health Promotion Board [3], [3] Internet sources [2]. Nutritional information of all food items (as prepared and ready-to-eat) was calculated by adding fat (oil), and sodium (salt) and/or garnishes such as garlic to raw ingredients. Dashes denote values not listed on nutritional labels or in databases. Carbs = carbohydrate; Sat Fat = saturated fat; Chol = cholesterol; B12 = Vitamin B12.

Table S5: Vegetarian-Novel (Veg-New) Diet

	Item	Serving	Calories	Carbs (g)	Protein (g)	Total Fat (g)	Sat Fat (g)	Chol (mg)	Sodium (mg)	Sugar (g)	Dietary Fibre (g)	B12 (ug)	Calcium (mg)	Potassium (mg)	Magnesium (mg)	Phosphorus (mg)	Zinc (mg)	Iron (mg)
**Breakfast**	White bread[1]	2 slices, 95g	237.50	47.50	6.48	2.16	0	0	453.41	4.32	2.18	0	86.36	111.15	34.11	97.85	0.84	4.64
	Olive-based margarine[1,2]	7g	35.14	0.04	0.02	3.95	0.77	0	18.69	0	0	0	0	1.19	0.07	0.21	0	0
	Plant-based egg omelette with dairy-free cheese[1,2,3]	115g	236.43	7.84	9.09	18.37	7.90	0	1549.09	0	0	0	4.94	46.96	14.21	19.64	0.14	0.23
	Bacon, meatless[1]	18g	78.49	0.73	1.52	8.39	4.40	0	383.80	0	0.34	0	3.16	22.14	2.48	9.10	0.06	0.32
	Ketchup[1]	15g	15.15	4.11	0.16	0.02	0	0	136.05	3.20	0.05	0	2.25	42.15	1.95	3.90	0.03	0.05
	Low fat milk[1]	200ml	80.00	9.10	6.34	2.02	1.27	8.00	102.00	9.10	0	0.70	226.00	314.00	22.00	170.00	0.74	0.06
**Morning Snack**	Black coffee[1]	250ml	5.00	0.85	0.25	0	0.01	0	10.00	0	0	0	10.00	75.00	10.00	7.50	0.03	0.10
	Plant-based beef jerky[1]	35g	112.35	10.00	12.50	1.25	0	0	800.10	5.01	0	0	0	0	6.72	133.56	0.69	1.50
**Lunch**	Plant-based burger with low fat cheese[1,3]	252g	492.17	32.66	33.66	26.09	9.22	15.40	1838.65	12.26	8.61	0.50	414.49	457.64	34.18	264.70	1.91	4.17
	French fries[1,3]	71g	221.50	29.37	2.43	10.44	1.66	0	149.28	0	2.43	0	12.80	320.21	26.28	88.60	0.36	0.58
	Soda[1,3]	620ml	210.00	56.00	0	0	0	0	55.00	56.00	0	0	6.20	31.00	0	55.80	0.56	0.12
**Afternoon Snack**	Strawberries[1]	100g	32.00	7.68	0.67	0.30	0.02	0	1.00	4.89	2.00	0	16.00	153.00	13.00	24.00	0.14	0.41
	Plant-based beef jerky[1]	35g	112.35	10.00	12.50	1.25	0	0	800.10	5.01	0	0	0	0	6.72	133.56	0.69	1.50
**Evening Meal**	Plant-based chicken tenders[1,3]	104g	193.13	15.70	13.73	9.83	1.65	0	1226.98	0	0	0	0.50	0.16	17.02	335.00	0.70	2.36
	Ketchup[1]	15g	15.15	4.11	0.16	0.02	0	0	136.05	3.20	0.05	0	2.25	42.15	1.95	3.90	0.03	0.05
	Stir-fried broccoli[1,2]	93g	65.56	9.08	2.78	2.89	2.24	0	222.30	1.42	2.24	0	39.35	296.14	17.86	66.30	0.40	0.69
	White rice[1]	60g	77.40	16.79	1.60	0.17	0.05	0	147.00	0.03	0.24	0	6.00	21.00	7.20	25.80	0.29	0.71
**Dessert**	Vegan coconut ice cream[1]	80g	234.40	20.00	2.61	18.26	11.30	0	4.00	14.78	1.76	0	17.60	174.60	29.20	73.20	0.65	1.26
	Peach[1]	150g	63.00	15.20	1.36	0.41	0.03	0	19.50	12.60	2.25	0	6.00	183.00	12.00	33.00	0.35	0.51
**Total**			**2516.7**	**296.8**	**107.8**	**105.8**	**40.5**	**23.4**	**8053.0**	**131.8**	**22.1**	**1.2**	**853.9**	**2291.5**	**256.9**	**1545.6**	**8.6**	**19.3**

Sources: [1] United States Department of Agriculture [1], [2] Health Promotion Board [3], [3] Internet sources [2,6], [4] On-pack nutritional labels. Nutritional information of all food items (as prepared and ready-to-eat) was calculated by adding fat (oil), and sodium (salt) and/or garnishes such as garlic to raw ingredients. Dashes denote values not listed on nutritional labels or in databases. Carbs = carbohydrate; Sat Fat = saturated fat; Chol = cholesterol; B12 = Vitamin B12.

Table S6: Vegan-Traditional (Vegan-Trad) Diet

	Item	Serving	Calories	Carbs (g)	Protein (g)	Total Fat (g)	Sat Fat (g)	Chol (mg)	Sodium (mg)	Sugar (g)	Dietary Fibre (g)	B12 (ug)	Calcium (mg)	Potassium (mg)	Magnesium (mg)	Phosphorus (mg)	Zinc (mg)	Iron (mg)
**Breakfast**	White bread[1]	1 slice, 48g	118.75	23.75	3.24	1.08	0	0	226.70	2.16	1.09	0	43.18	55.58	17.06	48.93	0.42	2.32
	Peanut butter[1]	15g	105.00	3.00	4.50	8.00	1.50	0	19.95	1.00	1.01	0	10.05	100.05	25.5	55.35	0.42	0.36
	Plant-based egg omelette with black beans[1,2]	139g	273.27	13.67	13.52	18.36	0.73	0	427.59	0.16	4.35	0	17.82	220.06	42.68	85.84	0.69	1.27
	Soymilk[3]	300ml	167.75	23.49	7.93	4.88	0.82	0	42.70	23.18	1.83	1.17	30.50	298.90	64.50	146.40	0.61	1.22
**Morning Snack**	Strawberries[1]	100g	32.00	7.68	0.67	0.30	0.02	0	1.00	4.89	2.00	0	16.00	153.00	13.00	24.00	0.14	0.41
	Black coffee[1]	250ml	5.00	0.85	0.25	0	0.01	0	10.00	0	0	0	10.00	75.00	10.00	7.50	0.03	0.10
**Lunch**	Bean burrito with hummus and dairy-free cheese[1,2,3]	266g	489.65	70.71	14.87	17.22	8.10	0	1417.05	9.25	8.96	0	167.85	547.15	71.05	339.70	1.76	4.85
	French fries[1,2]	31g	96.71	12.82	1.06	4.56	0.73	0	65.18	0	1.06	0	5.59	139.81	11.47	38.68	0.16	0.25
	Soda[1,2]	620ml	210.00	56.00	0	0	0	0	55.00	56.00	0	0	6.20	31.00	0	55.80	0.56	0.12
**Afternoon Snack**	Pumpkin seeds[1]	30g	172.20	4.41	8.94	14.70	2.56	0	5.40	0.39	1.95	0	15.60	236.40	165.00	351.00	2.29	2.42
	Soymilk[3]	300ml	167.75	23.49	7.93	4.88	0.82	0	42.70	23.18	1.83	1.17	30.50	298.90	64.50	146.40	0.61	1.22
**Evening Meal**	Tofu[1,3]	157g	191.50	3.80	15.20	12.09	1.28	0	804.50	0	1.95	0	114.48	474.16	48.62	178.50	1.65	2.06
	Black beans[1]	81g	105.60	18.97	7.09	0.43	0.11	0	383.60	0.26	6.96	0	21.72	284.04	56.01	112.00	0.90	1.68
	Stir-fried broccoli[1,2]	94g	79.46	9.06	2.78	4.31	0.39	0	28.30	1.42	2.24	0	39.20	296.10	17.85	66.30	0.39	0.69
	White rice[1]	25g	32.25	7.00	0.67	0.07	0.02	0	61.25	0.01	0.10	0	2.50	8.75	3.00	10.75	0.12	0.30
**Dessert**	Coconut yogurt[1]	50g	32.00	5.60	0.40	1.00	1.00	0	6.00	2.00	1.20	0	0	28.00	12.5	17.5	0.2	0
	Peach[1]	150g	63.00	15.20	1.36	0.41	0.03	0	19.50	12.60	2.25	0	6.00	183.00	12.00	33.00	0.35	0.51
	Pumpkin seeds[1]	30g	172.20	4.41	8.94	14.70	2.56	0	5.40	0.39	1.95	0	15.60	236.40	165.00	351.00	2.29	2.42
**Total**			**2514.1**	**303.9**	**99.3**	**107.0**	**20.7**	**0**	**3621.8**	**136.9**	**40.7**	**2.3**	**552.8**	**3666.3**	**799.7**	**2068.6**	**13.6**	**22.2**

Sources: [1] United States Department of Agriculture [1], [2] Internet sources [2,6], [3] Health Promotion Board [3]. Nutritional information of all food items (as prepared and ready-to-eat) was calculated by adding fat (oil), and sodium (salt) and/or garnishes such as garlic to raw ingredients. Dashes denote values not listed on nutritional labels or in databases. Carbs = carbohydrate; Sat Fat = saturated fat; Chol = cholesterol; B12 = Vitamin B12.

Table S7: Vegan-Novel (Vegan-New) Diet

	Item	Serving	Calories	Carbs (g)	Protein (g)	Total Fat (g)	Sat Fat (g)	Chol (mg)	Sodium (mg)	Sugar (g)	Dietary Fibre (g)	B12 (ug)	Calcium (mg)	Potassium (mg)	Magnesium (mg)	Phosphorus (mg)	Zinc (mg)	Iron (mg)
**Breakfast**	White bread[1]	2 slices, 95g	237.50	47.50	6.48	2.16	0	0	453.41	4.32	2.18	0	86.36	111.15	34.11	97.85	0.84	4.64
	Coconut spread[1]	14g	35.50	7.10	0	0.71	0.71	0	46.15	4.97	0	0	0	13.15	1.85	5	0.03	0
	Plant-based egg omelette[1,2]	85g	143.93	1.83	9.09	11.07	1.65	0	1279.09	0	0	0	4.94	42.76	7.71	15.84	0.14	0.23
	Bacon, meatless[1]	43g	275.67	8.53	11.95	22.62	10.46	0	1024.85	3.12	1.23	0	10.18	84.84	19.03	116.68	0.67	1.67
	Ketchup[1]	15g	15.15	4.11	0.16	0.02	0	0	136.05	3.20	0.05	0	2.25	42.15	1.95	3.90	0.03	0.05
	Soymilk[2]	300ml	167.75	23.49	7.93	4.88	0.82	0	42.70	23.18	1.83	1.17	30.50	298.90	64.50	146.40	0.61	1.22
**Morning Snack**	Plant-based beef jerky[1]	30g	96.3	8.57	10.71	1.07	0	0	685.8	4.29	0	0	0	0	5.76	114.48	0.59	1.29
	Black coffee[1]	250ml	5.00	0.85	0.25	0	0.01	0	10.00	0	0	0	10.00	75.00	10.00	7.50	0.03	0.10
**Lunch**	Plant-based burger with dairy-free cheese[1,3]	268g	610.82	50.81	24.76	35.86	19.10	0	2014.85	7.54	3.95	0	202.33	415.28	30.88	89.90	0.76	5.11
	French fries[1,2]	31g	96.71	12.82	1.06	4.56	0.73	0	65.18	0	1.06	0	5.59	139.81	11.47	38.68	0.16	0.25
	Soda[1,2]	620ml	210.00	56.00	0	0	0	0	55.00	56.00	0	0	6.20	31.00	0	55.80	0.56	0.12
**Afternoon Snack**	Plant-based beef jerky[1]	30g	96.30	8.57	10.71	1.07	0	0	685.80	4.29	0	0	0	0	5.76	114.48	0.59	1.29
	Unsalted nuts[1]	8g	46.96	1.70	1.95	3.97	0.62	0	0.48	0.39	0.67	0	4.64	50.72	14.24	29.04	0.22	0.13
**Evening Meal**	Plant-based chicken tenders[1,3]	104g	193.13	15.70	13.73	9.83	1.65	0	1226.98	0	0	0	0.50	0.16	17.02	335.00	0.70	2.36
	Ketchup[1]	15g	15.15	4.11	0.16	0.02	0	0	136.05	3.20	0.05	0	2.25	42.15	1.95	3.90	0.03	0.05
	Stir-fried broccoli[1,3]	97g	98.05	9.11	2.78	6.76	5.46	0	222.30	1.42	2.24	0	39.39	296.14	17.86	66.30	0.40	0.69
	White rice[1]	25g	32.25	7.00	0.67	0.07	0.02	0	61.25	0.01	0.10	0	2.50	8.75	3.00	10.75	0.12	0.30
**Dessert**	Strawberries[1]	100g	32.00	7.68	0.67	0.30	0.02	0	1.00	4.89	2.00	0	16.00	153.00	13.00	24.00	0.14	0.41
	Peach[1]	150g	63.00	15.20	1.36	0.41	0.03	0	19.50	12.60	2.25	0	6.00	183.00	12.00	33.00	0.35	0.51
**Total**			**2471.2**	**290.7**	**104.4**	**105.4**	**41.3**	**0**	**8166.4**	**133.4**	**17.6**	**1.2**	**429.6**	**1987.9**	**272.1**	**1308.5**	**7.0**	**20.4**

Sources: [1] United States Department of Agriculture [1], [2] Internet sources [2,5,6], [3] Health Promotion Board [3]. Nutritional information of all food items (as prepared and ready-to-eat) was calculated by adding fat (oil), and sodium (salt) and/or garnishes such as garlic to raw ingredients. Dashes denote values not listed on nutritional labels or in databases. Carbs = carbohydrate; Sat Fat = saturated fat; Chol = cholesterol; B12 = Vitamin B12.

References

1. United States Department of Agriculture. FoodData Central. Available online: http://fdc.nal.usda.gov/ (accessed on 10 March 2021).
2. McDonald's. Nutrition Calculator. Available online: https://www.mcdonalds.com/us/en-us/about-our-food/nutrition-calculator.html (accessed on 13 April 2021).
3. Health Promotion Board. Energy & Nutrient Composition of Food. Available online: https://focos.hpb.gov.sg/eservices/ENCF/ (accessed on 12 March 2021).
4. Beyond Meat. Our Products. Available online: https://www.beyondmeat.com/products/ (accessed on 9 March 2021).
5. Gardein. Gardein Seven Grain Crispy Tenders. Available online: https://www.gardein.com/chickn-and-turky/classics/seven-grain-crispy-tenders (accessed on 10 March 2021).
6. JUST Egg. JUST Egg | Made from plants. Available online: https://www.ju.st/en-us/products/consumer/egg/egg (accessed on 15 March 2021).
